# Silencing of *SlDRB1* gene reduces resistance to tomato yellow leaf curl virus (TYLCV) in tomato (*Solanum lycopersicum*)

**DOI:** 10.1080/15592324.2022.2149942

**Published:** 2022-12-01

**Authors:** Xin Huang, Jianming Wei, Dan Wu, Na Mi, Sili Fang, Yao Xiao, Yunzhou Li

**Affiliations:** Department of Plant Pathology, College of Agriculture, Guizhou University, Guiyang, China

**Keywords:** Tomato, VIGS, TYLCV, double-stranded RNA-binding proteins1(DRB1), antiviral

## Abstract

Double-stranded RNA-binding proteins are small molecules in the RNA interference (RNAi) pathway that form the RNAi machinery together with the Dicer-like protein (DCL) as a cofactor. This machinery cuts double-stranded RNA (dsRNA) to form multiple small interfering RNAs (siRNAs). Our goal was to clarify the function of *DRB* in tomato resistant to TYLCV. In this experiment, the expression of the *SlDRB1* and *SlDRB4* genes was analyzed in tomato leaves by qPCR, and the function of *SlDRB1* and *SlDRB4* in resistance to TYLCV was investigated by virus-induced gene silencing (VIGS). Then, peroxidase activity was determined. The results showed that the expression of *SlDRB1* gradually increased after inoculation of ‘dwarf tomato’ plants with tomato yellow leaf curl virus (TYLCV), but this gene was suppressed after 28 days. Resistance to TYLCV was significantly weakened after silencing of the *SlDRB1* gene. However, there were no significant expression differences in *SlDRB4* after TYLCV inoculation. Our study showed that silencing *SlDRB1* attenuated the ability of tomato plants to resist virus infection; therefore, *SlDRB1* may play a key role in the defense against TYLCV in tomato plants, whereas *SlDRB4* is likely not involved in this defense response. Taken together, These results suggest that the *DRB* gene is involved in the mechanism of antiviral activity.

## Introduction

Tomato (*Solanum lycopersicum*) is an important vegetable crop in the Solanaceae and is also one of the most important vegetables in the world.^1^ Many people enjoy consuming tomatoes for their nutritional benefits, such as lycopene, flavonoids, and *β*-carotene.^2^ However, various biotic and abiotic stresses, especially tomato yellow leaf curl virus (TYLCV), threaten tomato production. In China, TYLCV has become an important viral disease that has threatened tomato production since 2002.^3^

TYLCV is a single-stranded circular DNA virus that belongs to the genus *Begomovirus*. TYLCV can infect a variety of crops, including tomato, pepper, and common bean.^4–6^ After infection with TYLCV, tomato plants show yellowing and curling of the leaf margins and an inhibition of flowering and fruiting, which results in a decline in production.The breeding of resistant varieties is one of the means of prevention. Among them, the construction of CRISPR/Cas9 system is gradually stabilizing. Tashkandi et al. showed that Cas9-single guide RNA targeting the shell protein (CP) or replicase (Rep) sequence encoded by TYLCV genome can effectively interfere with the virus.^7^

Currently, the genetic/molecular markers *Ty-1, Ty-2, Ty-3, Ty-4, Ty-5*, and *Ty-6* for resistance to TYLCV have been studied in tomato.^8–12^ Among them, *Ty-1*/*Ty-3* is a pair of alleles encoding *RDR γ* protein,^13^ which generates resistance to TYLCV by increasing cytosine methylation of the viral genome.^14^ Therefore, the study of gene expression changes during resistance to TYLCV and the selection and breeding of new resistant varieties have been important tools for controlling viral spread in recent years. Analysis of the role of resistance-related genes in defense against viral diseases is important for improving the resistance to TYLCV in tomato.

Double-stranded RNA-binding (DRB) proteins are key proteins in the RNA silencing signaling pathway that respond to a range of stimuli, such as plant hormones, biotic stresses, and abiotic stresses.^15–17^*DRB* genes are widely present in eukaryotic cells, bacteria, and viruses; are involved in RNA processing; are important factors in the RNA interference pathway; and may play an important role in disease resistance. The first reported case of DRB identification was in *Arabidopsis*^18^ and involved the *DRB1* gene, also known as *HYL1* which is the longest studied gene in *Arabidopsis*.^19^ The *DRB1* gene regulates plant disease resistance through the jasmonic acid pathway^20^and plays a role in abiotic stresses. Additionally, *drb1* mutants have the highest tolerance to cadmium (Cd) stress, and studies suggest that *Arabidopsis* has a complex miRNA-directed molecular response to Cd stress.^21^ There are eight members of the *DRB* gene family in tomato, and although the functional role of *DRBs* in tomato is still unreported, some studies have shown that almost all *DRB* family members have positive effects on plant disease resistance.^22^ However, the function of DRB is still not clear in tomato antiviral, especial in tomato against TYLCV. Therefore, in this study, qRT‒PCR was used to analyze the expression of *DRB* genes under abiotic stress in tomato plants inoculated with TYLCV at different time periods. Functional analysis of the identified *SlDRB1* and *SlDRB4* genes were performed in virus resistance by the VIGS technique to provide an in-depth investigation of the biological functional basis of these genes in tomato. This study provides a theoretical reference for investigations into the biological function of these genes and their expression in tomato.

## Materials and methods

### Plant materials and growth conditions

The test material used in this study was the ‘Dwarf Tomato’ variety, and the tomato seeds were soaked in warm broth at 55°C with 5–6 volumes of water. After soaking for 15 min, the seeds were planted in sterilized soil substrate after the temperature had cooled. The seeds were placed in a greenhouse set to 12 h of light (25°C)/12 h of darkness (18°C) and 60%-70% relative humidity. When the tomato plants grew to five leaves and one heart, the leaves were treated with the control and experimental treatments.

### Biological information analysis of SlDRB1

The sequences of the *SlDRB1* (Accession *No*. Solyc04g076420) and *SlDRB4* (Accession *No*. Solyc03g118950) genes were obtained from the tomato database (https://solgenomics.net), and the primers SlDRB1-F, SlDRB1-R, SlDRB4-F, and SlDRB4-R were designed using primer 6 and synthesized by Bioengineering (Shanghai) Co. Tomato leaf RNA was extracted, and the target fragments were amplified by RT‒PCR to obtain full-length SlDRB1 and SlDRB4.

The sequencing results were compared via BLAST by logging into the NCBI database to obtain the homologous amino acid sequences of SlDRB4 and SlDRB1 proteins. The physical and chemical properties of tomato DRB proteins were analyzed using Protparam software, an online tool provided by ExPasy (https://web.expasy.org/protparam/). SOPMA 2.0 software was used (https://npsa-pbil.ibcp.fr/cgi-bin/npsa- automat.plpage = npsa-sopma.html) to predict the secondary structure and analyze the α-helix, irregularly coiled and extended chains. SWISS-MODEL protein homology was used to build the membrane to predict the tertiary structure of the SlDRB1 and SlDRB4 protein.

### Abiotic stress treatment

Tomato plants with uniform growth were selected, and solutions with 20.0% polyethylene glycol-6000(PEG-6000), 0.50 mmol-L^−1^ salicylic acid(SA), 0.10 mmol-L^−1^ benzothiadiazole(BTH), and 300 mmol-L-1 NaCl were prepared. One hundred milliliters of the solution was used to submerge the tomato roots in the experimental group and 100 ml of sterile water was used in the control group. Total RNA was extracted from tomato leaves at 0 h, 3 h, 6 h, 12 h, and 24 h, and cDNA was reverse transcribed. Gene expression differences in the *SlDRB4* gene after abiotic stress treatments (PEG, BTH, SA, and NaCl) were detected by qPCR (Figure 2).

**Figure 1.**
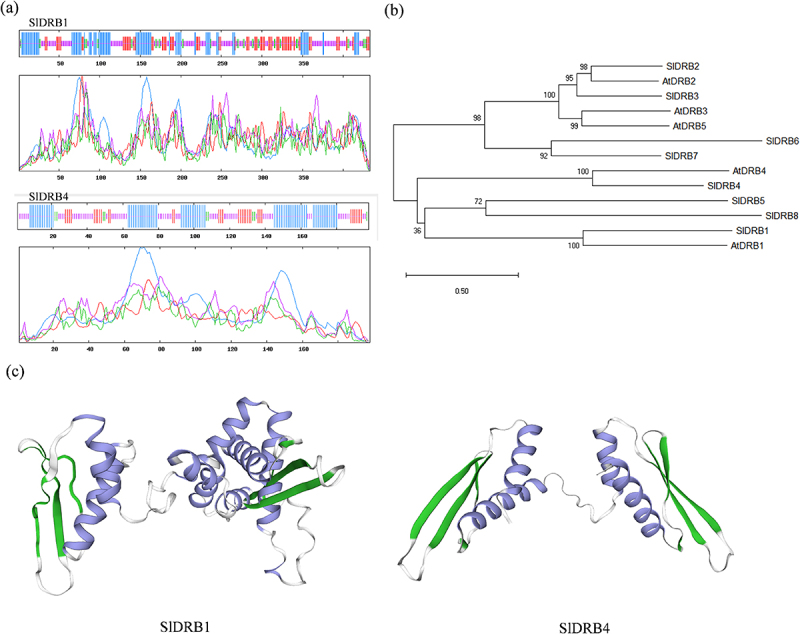
**Structure prediction and phylogenetic analysis of SlDRB1 and SlDRB4**.(a) Secondary structure prediction of SlDRB1 and SlDRB4 proteins; (b) Phylogenetic tree of SlDRB1, SlDRB4 and other proteins from *Arabidopsis thaliana* (ATDRB1:AT1G09700; ATDRB2:AT2G28380; ATDRB3:AT3G26932; ATDRB4:AT3G62800; ATDRB5:AT5G41070) and *Solanum lycopersicum* (SlDRB2:Solyc11g069460.1.1; SlDRB3:Solyc05g056100.2.1; SlDRB5:Solyc03g118950.1.1; SlDRB6:Solyc01g009190.1.1; SlDRB7:Solyc01g056600.2.1; SlDRB8:Solyc02g091460.2.1); (c) Prediction of the tertiary structure of SlDRB1 and SlDRB4 proteins.

### TYLCV infection

TYLCV-infected clones were obtained and transferred into *Agrobacterium tumefaciens* GV3101. An inoculum (200 mmol-L-1 acetosyringone, 10 mmol-L-1 MgCl, and 10 mmol-L-1 MES) was prepared to suspend the bacteria to OD_600_ values between 0.6 and 0.8^22^. The inoculum was placed on 6-week-old tomato plants in the dark for 2 h, while 1 ml of inoculum was slowly injected into the lower epidermis of tomato leaves until the leaves were filled with inoculum following the inoculation method described by Li et al (2017). Each treatment was replicated three times, and 0.50 g of leaf tissue was snap frozen in liquid nitrogen at 0 days, 7 days, 14 days, 24 days and 28 days and stored at −80°C.

### *Functional analysis of* SlDRB4 and SlDRB1 *based on the VIGS method*

Virus-mediated silencing vectors were constructed, partial SlDRB1 and SlDRB4 sequences were obtained by VIGS prediction from the tomato database, and the enzymatic cleavage sites *EcoRI* (5’ end) and *KpnI* (3’ end) were designed into the pTRV2 vector. Plasmids TRVl, TRV2-*SlDRB1*, TRV2-*SlDRB4*, and TRV2-*PDS* were transferred into *Agrobacterium* tumefaciens GV3101, and the bacterial solutions of TRVl and TRV2 recombinant plasmids were mixed at a 1:1 ratio (v:v) and incubated in the dark at room temperature for 3 h. *Agrobacterium* cultures were directly injected into 3-week-old tomato leaves using a 1 mL syringe, and a total of 40 infiltrated seedlings were obtained.^23,24^ As a positive control, tomato leaves injected with TRV:*PDS* showed significant whitening symptoms at about two weeks, with the more pronounced whitening the higher the degree of inhibition.

### Physiological and biochemical measurements

Reactive oxygen (ROS) can resist the invasion of viruses, and plants have a well-developed reactive oxygen scavenging system, mainly composed of SOD, CAT, POD, APX and other antioxidant enzymes. If these reactive oxygen species are not removed immediately, the host will be harmed, so the level of antioxidant enzymes in the host can reflect the degree of pathogen attack to a certain extent. Peroxidase (POD) activity was determined by an increase in absorbance at 470 nm caused by the oxidation of guaiacol; superoxide dismutase (SOD) activity was determined by the inhibition of the photochemical reduction of nitrotetrazolium blue chloride(NBT) at 560 nm; and catalase (CAT) activity was determined by a reduction in hydrogen peroxide extinction, i.e., a decrease in absorbance at 240 nm. SOD is responsible for converting excess reactive oxygen species into hydrogen peroxide, and then CAT and POD convert hydrogen peroxide; into water and oxygen in time.Ascorbate peroxidase (APX) activity was determined by the decrease in absorbance at 290 nm after ascorbate oxidation.^23,25^

### Quantitative real-time polymerase chain reaction (RT‒qPCR) analysis of gene expression

Real-time fluorescence quantitative PCR is an important technique for studying gene expression^26^ and plays an important role in analyzing gene transcript levels at different developmental periods, between different tissues and under different stress conditions. Seedlings with consistent growth to the 5–6 true leaf stage were selected for TYLCV-infected clonal inoculation, and tissue from the third true leaf was sampled at different treatment times and stored in a − 80°C refrigerator after quick freezing with liquid nitrogen. Leaf DNA was extracted using the cetyltrimethyl-ammonium bromide (CTAB) method, and actin was used as a control for quantitative analysis. Total RNA was extracted, and reverse transcribed cDNA was diluted as a template. The reaction procedure was as follows: predenaturation at 95°C for 30s; 40 cycles of denaturation at 95°C for 5 s followed by annealing at 60°C for 20s; and melting curve analysis at 95°C for 0 s, 65°C for 15s and 95°C for 0 s. The reaction was carried out in a fluorescent quantitative PCR instrument (CFX96TM Real-time System, Bio-Rad, USA) for quantitative analysis. The relative amount of gene expression was calculated using the 2^−ΔΔCt^ method with three replicates per treatment,^27^ using *SlEF1α* as an internal reference gene^28^

## Statistical analysis

Statistical analysis was performed using one-way ANOVA in SPSS 20.0 software. Differences in the expression of *SlDRB1* and *SlDRB4* after TYLCV infection were evaluated at a p value less than 0.05 using Duncan’s multiple range test for significance of differences between multiple samples in SPSS. A t test was used to compare the differences in viral content between the experimental and control groups with experimental *SlDRB4*, silenced *SlDRB1*, negative control (pTRV1 + pTRV2) and positive control (pTRV1 + pTRV2: *PDS*) plants (*p* < .05). Three replicates were used for each sample, and the results obtained are all expressed as the mean ± standard deviation (SD).

## Results

### Sequence and phylogenetic analyses of SlDRB4 and SlDRB1

Sequence analysis showed that the full length open reading frames of *SlDRB4* and *SlDRB1* are 867 bp and 1302 bp, respectively, and SlDRB1 encodes a protein with 566 amino acids and a predicted molecular weight of 45.84 kDa. The results of protein secondary structure prediction and analysis showed that SlDRB1 contains α-helix (27.02%), *β*-turn (8.78%), irregularly coiled (40.42%) and extended chain domains. The SlDRB4 protein consists of 198 amino acids and has a molecular weight of 22.44 kDa. A total of 39.39% of the secondary structure is composed of α-helices, 4.55% of β-folds, 38.89% of irregular curls and 17.17% of extension chains (Figure 1(a)). In addition, the tertiary structures of SlDRB1 and SlDRB4 were constructed by Phyer 2. 0.

**Figure 2.**
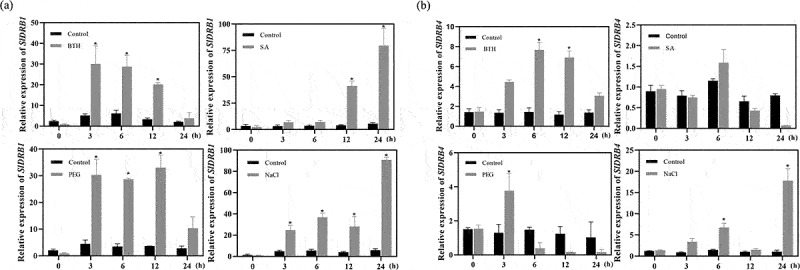
**Relative expression of the SlDRB1 and SlDRB4 genes in leaves under different stress treatments**.(a) and (b) 20% PEG-6000 treatment, 0.50 mmol·L^−1^ SA treatment, 0.10 mmol·L^−1^ BTH treatment, and 300 mmol·L^−1^ NaCl treatment.The assays for each treatment consisted of three biological replicates, and different lowercase letters indicate significant differences at the 0.05 level.

DRB proteins were first identified in *Arabidopsis*, and the similarities with SlDRB1 and SlDRB4 were further analyzed by constructing a phylogenetic tree. As shown in Figure 1(b), SlDRB1 shares more homology with AtDRB1, also known as HYL1, in *Arabidopsis*. A study showed that AtDRB1 is highly phosphorylated in *mpk3* mutants, while AtMPK3 is a negative regulator of AtDRB1 protein stability2^7^. Transgenic plants of HYL1 (AtDRB1) in *Arabidopsis thaliana* can regulate the necrotrophic pathogen gray mold (*Botrytis cinerea*) through the JA signaling pathway.^20^SlDRB4 showed more homology to AtDRB4 in the phylogenetic tree. One study demonstrated that AtDRB4 is involved in the turnip yellow mosaic virus (TYMV) antiviral response and that this protein is required for TYMV-derived small RNA production.^29^ These results suggest that DRB4 has a negative effect on the accumulation of viral capsid proteins. Overall, SlDRB1 and SlDRB4 may be involved in abiotic stress or pathogen infection responses.

### Expression of the SlDRB4 and SlDRB1 genes in plants under abiotic stress

The expression of the *SlDRB1* and *SlDRB4* genes was detected by qPCR after PEG, BTH, SA, and NaCl treatments (Figure 2). Total RNA was extracted from tomato leaves at 0 h, 3 h, 6 h and 12 h, and reverse transcribed cDNA was analyzed by real-time fluorescence quantitative PCR. *SlDRB1* gene expression showed an increasing trend under SA and NaCl stress, while *SlDRB4* gene expression increased only under NaCl stress but decreased under BTH, SA and drought stress treatments. The results indicated that the expression of *SlDRB1* and *SlDRB4* was induced under abiotic stress.

### Expression analysis of SlDRB1 and SlDRB4 after TYLCV inoculation

**Figure 3.**
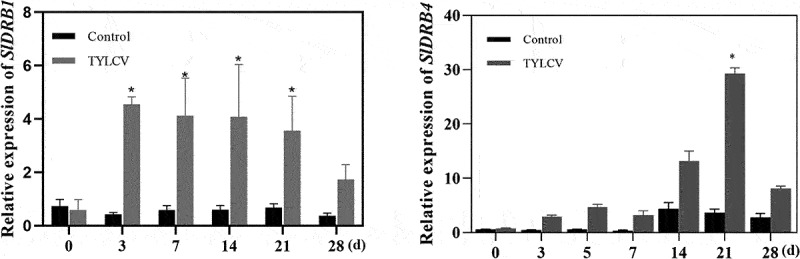
**Relative transcript expression of *SlDRB1* and *SlDRB4* inoculated with TYLCV for different time periods**.(a and b) Expression profiles of the *SlDRB1* and *SlDRB4* genes in response to TYLCV. The reference gene was *SlEF1α*, and the assays for each treatment consisted of three biological replicates. Data are the means ± standard errors of three independent experiments. Differences between time-course sampling points were assessed using SSPS. *p < .05, n = 3.

In this study, we analyzed the expression of the *SlDRB1* and *SlDRB4* genes in tomato leaves. After inoculation with TYLCV, the expression of the *SlDRB1* gene increased in the leaves over time compared with the control (Figure 3(a)). The *SlDRB4* gene reached its highest value at 21 day of inoculation, after which the expression decreased (Figure 3(b)). *SlDRB4* expression was higher than *SlDRB1* expression in both groups after TYLCV treatment, indicating that *SlDRB4* was more highly induced. These results indicate that TYLCV induces the expression of both the *SlDRB1* and *SlDRB4* genes.

### *Effect of silencing* SlDRB4 *and* SlDRB1 *on TYLCV infection*

**Figure 4.**
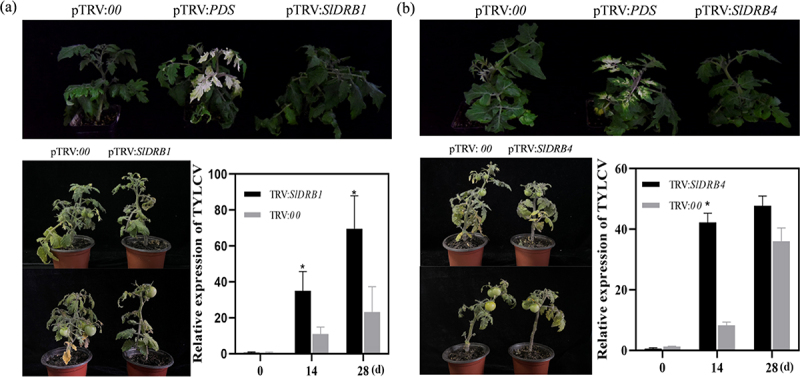
**VIGS of *SlDRB1* reduced the tolerance of plants to TYLCV**.(a) and (b) At the top of the figure, the phenotypes of SlDRB-silenced, negative control pTRV:*00* and positive control pTRV:*PDS* plants are shown. The figure on the left shows the phenotypes of TRV:*00*, TRV:*SlDRB1*, and TRV:*SlDRB4* at 14 days and 28 days after TYLCV inoculation. The figure on the right shows the relative expression of TYLCV in TRV:*SlDRB1*, TRV:*SlDRB4* and TRV:00 plants at different time periods.

To analyze the disease resistance of SlDRB1 and SlDRB4 proteins in tomato, virus-mediated gene silencing was used to inoculate leaves with *SlDRB1* and *SlDRB4* gene silencing and control (TRV:*00*) solutions. After the infection of TRV:PDS, plants showed whitening symptoms, indicating that the gene had been successfully silenced (Figure 4(a,b)). As shown in the lower left panels of Figures 4(a-b), representing the plant phenotypes at day 14 and 28 after TYLCV inoculation, *SlDRB1* showed more curling of tomato leaf tips and a greater decrease in fruit set at day 28 after silencing compared to the control. To further verify the silencing effect on the regulation of TYLCV, real-time fluorescence quantitative PCR was used to detect viral expression. Quantitative analysis showed that the accumulation of TYLCV was elevated at 28 d relative to the control, whereas silencing of the *SlDRB4* gene did not produce significantly different results compared to the control.

### The activity of antioxidant enzymes was detected

**Figure 5.**
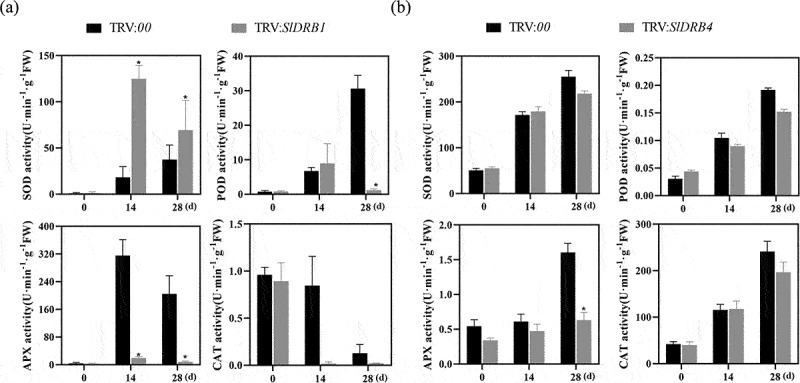
**Changes in antioxidant enzyme activity after virus inoculation**.(a)Relative expression levels of POD, SOD, APX and CAT in *SlDRB1*-silenced plants (* denotes *P* < .05); (b) Relative expression levels of POD, SOD, APX and CAT in *SlDRB4*-silenced plants (* denotes *P* < .05).

Reactive oxygen species (ROS) have certain immune and signal transduction functions in plants. The activities of several important components of the enzymatic defense system, POD, SOD, CAT, and APX, were further determined. Compared to the control (TRV:*00*), antioxidant enzyme activities in TYLCV-inoculated tomato leaves all changed significantly with disease progression. The *SlDRB4*-silenced plants showed similar and nonsignificant changes in SOD, CAT, and POD activities compared to the control, except for APX (Figure 5(b)). In contrast, the *SlDRB1*-silenced plants showed fewer changes in POD, CAT and APX, but not SOD, activities than the control (Figure 5(a)). Thus, virus inoculation resulted in different changes in antioxidant enzyme activities in tomato, which resulted in changes to ROS scavenging, the maintenance of ROS homeostasis in vivo and the avoidance of oxidative damage to membranes.

## Discussion

TYLCV is one of the important virus affecting tomato yield, and there are few means available to control TYLCV. One of the most effective methods is to improve TYLCV resistance by breeding disease-resistant varieties with disease resistance genes; currently, the most effective and widely used disease resistance genes are *Ty-1* to *Ty-6*.^10,11,30,31^ Therefore, mining unknown TYLCV resistance genes will be one of the main means of control in the future.

In plants, many antiviral defense mechanisms include RNAi pathways and innate immune system-mediated responses. One study designed three RNAi gene structures based on the outer coat protein (CP) and untranslated regions of the potato virus Y(PVY) genome and found that siRNAs generated from dsRNAs could activate the immune system in transgenic plants via the detection of degraded viral transcripts.^32^ In addition, many RNAi pathway-related proteins have been reported.^33^ For example, silencing of the SlDCL2 and SlDCL4 genes in tomato by VIGS technology disrupts the resistance of the Ty-1/Ty-3 allele to resistance to TYLCV.^34^ The *DRB* genes have been mostly studied in *Arabidopsis* and have been found to play an important role in the regulation of resistance to pathogenic infection in different species. Authors You et al. showed that in apple *MdDRB1* controls adventitious roots, leaf curvature and tree structure by regulating the transcript levels of miRNAs.^35^In addition, studies in rice have identified a previously unknown mechanism by which the key component OsDRB1 is hijacked by a viral protein for miRNA biogenesis, thereby enhancing the viral infection and pathogenesis in rice.^36^ In *Arabidopsis*, the interaction of DCL4 with DRB4 may be involved in the antiviral response.^37^ The DRB protein complex has antagonistic effects on RNase III activity and siRNA production in plants, thus affecting dsRNA processing.^38^

Phylogenetic analysis showed that SlDRB1 and SlDRB4 belong to the same branch as DRB1 and DRB4, respectively, in *Arabidopsis*,Functional studies of the DRB family have focused on the role of micro RNA (mi RNA) synthesis.^39^ It has been shown that mi RNA plays an important regulatory role in plant growth and development as well as in plant responses to abiotic stresses such as salt stress, drought, and heavy metal stress.^40,41^Therefore, in this study, the quantitative analysis of *SlDRB1* and *SlDRB4* adversity stresses by PEG, SA, Nacl, and BTH treatments showed that both *SlDRB1* and *SlDRB4* were induced to be expressed under stress, so it can be speculated that *DRBs* also plays an important role in tomato growth and development. In addition,DRB1 and DRB4 also play an important role in antiviral resistance.^15,22^ Therefore, we hypothesized that *SlDRB1* and *SlDRB4* might be involved in the defense response of tomato against TYLCV. In the present study, tomato plants were infected with TYLCV, thereby inducing the transcription of DRB1 and DRB4. Subsequently, tomato plants were silenced by the VIGS technique and inoculated with TYLCV to further investigate the functions of *SlDRB1* and *SlDRB4*. Both *SlDRB1* and *SlDRB4* were induced in tomato plants inoculated with TYLCV, but expression began to decrease after 21 days. Silencing of *SlDRB1* resulted in significantly higher accumulation of TYLCV compared to the negative control, indicating that silencing of this gene reduces the defense response of tomato plants to TYLCV. In contrast, after silencing the *SlDRB4* gene, the accumulation of TYLCV was not significantly different from that in the control, and it is speculated that the *SlDRB4* gene is not involved in the tomato defense response against TYLCV. Reactive oxygen species in plants are mainly derived from photosynthesis and respiration, and in green plants, chloroplasts are the main source of reactive oxygen species production. Silencing the *SlDRB1* gene decreases antioxidant enzyme activity, and some studies have reported that the DRB1 protein in *Arabidopsis* participates in the miRNA-regulated photomorphic building process^42^ Therefore, we hypothesized that silencing *SlDRB1* together with TYLCV infection affects reactive oxygen species production, as there was an increase in SOD enzyme activity at 14 days compared to the control, while POD, APX, and CAT, but not SOD, activities were lower than the control at 28 days.

Studies have shown that DRB family members are negatively regulated by photomorphogenesis, which affects their disease resistance.^22^ The DRB family is currently studied in the model plant Arabidopsis thaliana, with fewer reports in other species. The most studied proteins with structural domains in the RNAi interference pathway with disease resistance aspects are the Dicer-like (DCL) family, which have a synergistic relationship with DRB family members and have functional similarities. DRB proteins assist DCL proteins to act together on miRNAs, which can bind to target gene mRNA molecules and act in transactivation by blocking mRNA translation or The DRB proteins assist DCL proteins to act together with miRNAs, which can bind to target gene mRNA molecules and regulate gene expression at the transcriptional level by blocking mRNA translation or cutting target genes,^43^ thereby regulating plant development. We speculate that DRB proteins may be regulated to affect plant disease resistance, which can be further verified by protein interactions.

In conclusion, our study found that *SlDRB1* plays a positive regulatory role in the defense process against TYLCV. It was speculated that SlDRB1 might regulate the RNAi pathway to resistance virus. This study will provide a reference for further research on the resistance mechanism of tomato plants to TYLCV.

## Data Availability

We have read and understood your journal’s policies, and we believe that neither the manuscript nor the study violates any of these. There are no conflicts of interest to declare.
